# Comparative analysis of maternal and neonatal outcomes in cases of vaginal breech births versus cases of vaginal cephalic births: a retrospective cohort study

**DOI:** 10.1007/s00404-025-08126-z

**Published:** 2025-07-22

**Authors:** Hanna Kriegs, Joachim Graf, Harald Abele, Claudia Plappert

**Affiliations:** 1 Section of Midwifery Science, Institute for Health Sciences, University Hospital Tübingen, Calwer Street 7, 72076 Tübingen, Germany; 2 Department for Women’s Health, University Hospital Tübingen, Calwer Street 7, 72076 Tübingen, Germany

**Keywords:** Vaginal breech birth, Vaginal cephalic birth, Maternal outcomes, Neonatal outcomes, Retrospective cohort study

## Abstract

**Purpose:**

This study examines whether the perinatal mortality rates (up to 7 days postpartum) of successful vaginal breech birth (VBB) align with those of vaginal cephalic birth (VCB) under the current practice of risk stratification and promotion of VBB in Germany. This study excludes births that did not result in vaginal breech birth, i.e., cases where a vaginal birth was attempted but discontinued.

**Methods:**

A retrospective cohort analysis of the 2021 German population dataset compared 1435 VBBs to 422,019 VCBs. Maternal and neonatal short-term outcomes were analyzed using Chi-squared and Mann–Whitney *U* tests.

**Results:**

*Main outcome:* No significant difference in perinatal mortality rates between VBB and VCB. *Other neonatal outcomes*: Mean arterial blood gas levels and mean APGAR levels were lower in the breech group. The need for resuscitation measures and transfers to the pediatric hospital were increased. *Maternal outcomes*: Births in the breech group received labor augmentation more frequently and had higher rates of episiotomies. They had lower rates of perineal tears and postpartum complications. The rates of hysterectomies and increased postpartum hemorrhage did not differ significantly.

**Conclusion:**

With thorough risk stratification and interdisciplinary expert management, perinatal mortality rates (up to 7 days postpartum) of VBBs align with VCBs. However, higher neonatal intervention rates in VBB highlight the need to ensure adequate resources and preparedness for postnatal support.

## What does this study add to the clinical work

**Table:** 

This paper evaluates the current breech birth system in Germany. The results show that with thorough risk stratification and interdisciplinary expert management, perinatal mortality rates of VBBs align with VCBs.	

## Introduction

### Background

Childbirth is a significant event for women and their families, where the mode of birth chosen can have a significant impact on maternal and neonatal short- and long-term outcomes. Although cephalic presentation is the most common, approximately 3–4% of full-term babies are born in the breech position [1]. In the past, advice on mode of delivery was strongly influenced by the results of the “Term Breech Trial”, which indicated an increased risk of adverse birth outcomes in vaginal breech births (VBB) [2]. Therefore, in many countries, delivery by cesarean section was usually advised in the case of breech presentation, which minimized the incidence of vaginal breech birth in clinical practice.

Recently, several studies have been published that question the necessity of cesarean delivery in breech presentation. These show that with strict risk stratification and appropriate expertise, the outcomes of VBB can be very similar to those of vaginal birth in the cephalic position (VCB) [3–6]. As a result, VBB is now supported by gynecologists and midwives after thorough risk screening.

### Management of breech presentation in Germany

From around 36 weeks of gestation, detailed counseling on mode of delivery is offered by midwives or obstetricians. The families are informed about the risks and procedures of both vaginal breech birth and cesarean section, advised on the choice of hospital, and given sufficient time to make an informed decision [1].

Referral to a specialized perinatal center is recommended for individualized counseling. Counseling on external cephalic version is also recommended from 37 weeks of gestation [7].

At the specialized clinic, risk selection for VBB includes fetal weight estimation, head circumference, head-to-abdomen ratio, amniotic fluid volume, placental location, and fetal position and attitude [1]. According to the AWMF guideline on cesarean section, when strict risk selection is applied, VBB is considered an equivalent alternative to c-section [7]. Along these recommendations, the c-section rate for breech presentation remains high in Germany. According to the 2021 IQTIG national report, 64.2% of term singleton breech births (*n* = 26.862) were delivered by planned c-section [8].

### Aims

This paper aims to evaluate whether the contemporary system effectively aligns the mortality of VBB with that of VCB, ensuring safe outcomes through structured prenatal care and management. A critical comparison of VBB and VCB offers a meaningful perspective on the outcomes of vaginal breech delivery. This paper aims to analyze maternal, fetal, and neonatal outcomes under current German practices, to evaluate whether these outcomes can achieve parity with cephalic deliveries. Such an outcome-based analysis contributes to empower women through evidence-based counseling, supporting autonomy and shared decision-making during pregnancy and childbirth. Midwives play a central role in this discourse, emphasizing the importance of individualized care, informed choice, and evidence-based practice in breech birth management, while caring for families during pregnancy and birth [6, 9, 10].

## Methods

### Study design

The study employed a retrospective cohort design to compare maternal, fetal, and neonatal outcomes between VBB and VCB. Data were sourced from the German Institute for Quality Assurance and Transparency in Health Care (IQTIG), which, as part of the mandatory cross-institutional quality assurance program, collects predefined outcome measures from all births in hospitals in Germany, covering approximately 98.5% of all births and thus representing a near-complete survey. This ensures a robust and comprehensive dataset for analysis.

### Study population and definition of subgroups

The study population consisted of 1435 breech and 422,019 cephalic singleton term births of not prepartum deceased babies from clinics all over Germany in the year 2021. Vacuum and forceps deliveries in cephalic presentations were excluded. Subgroups were defined based on fetal presentation, with further stratification by relevant maternal and neonatal characteristics to ensure comparability and control for confounding variables.

### Outcome variables

Outcome variables were selected to reflect the short-term consequences of the fetal presentation during vaginal birth on maternal and neonatal health. This selection was based on the predefined variables (quality indicators and performance measures) from the IQTIG dataset [11]. These included the use of labor-inducing agents, episiotomy rates, perineal tears, postpartum hemorrhage (PPH), hysterectomies, postpartum complications, and neonatal outcomes, such as arterial base excess, pH, APGAR scores, the need for resuscitation, transfer to NICU, mortality, and duration of labor.

### Definition of characteristics and outcome variables

“High-risk pregnancy/Findings in maternity” records include documented risk factors, such as pre-existing conditions (e.g., diabetes, hypertension), previous cesarean, infections, or abnormal findings in the maternity record (“Mutterpass”) as coded in the IQTIG dataset.

“Malformations/Illness of the newborn” refers to congenital anomalies or neonatal conditions documented at birth, such as chromosomal or cardiac defects. Only diagnoses available at discharge are included.

“Fetal death” timing is based on fetal heart activity at admission. The IQTIG system distinguishes antepartum (no heart activity at admission) versus intrapartum (heart activity at admission but absence at birth) stillbirths. Only intrapartum stillbirths were included in this study.

“Postpartum complications requiring treatment” are conditions treated during inpatient stay (e.g., uterine atony, retained placenta, infections). Complications occurring after discharge are not included in the dataset.

### Data analysis

Data were analyzed using Chi-squared tests and Mann–Whitney *U* tests to identify differences in outcomes between the two groups. A significance level of 0.05 was set for all statistical tests to determine significant group differences. All effect sizes were calculated with 95% confidence intervals. To address potential confounders, predefined characteristics (see Table 1) were compared between the breech and cephalic presentation groups using chi-squared, Mann–Whitney *U*, and *t *tests with a significance level of 0.1%.

**Table 1:** Maternal characteristics

Characteristics		VBB	VCB	Significance
		Number (%) or mean ± SD median	Number (%) or mean ± SD median	*p* value
Maternal age		32.12 ± 4.22	30.91 ± 5.07	< 0.0001
Parity		1.32 ± 1.07 1	1.38 ± 1.05 1	0.0068
High-risk pregnancy/findings in maternity records	Yes	*n* = 613 (42.7%)	*n* = 311,861 (73.9%)	< 0.0001
Gestational age (in days p.m.)		276.66 ± 7.92	278.67 ± 7.45	< 0.0001
Malformations/illness of the newborn	Yes	*n* = 5 (0.3%)	*n* = 682 (0.2%)	0.1536
Birthweight (in gram)		3253.45 ± 385.37 3250	3475.68 ± 436.83 3470	< 0.0001

*SD* standard deviation, *VBB* vaginal breech birth, *VCB* vaginal cephalic birth

### Ethical considerations

The data collection and analysis were carried out by the IQTIG. Through the proven practices, ethical standards and data confidentiality principles were adhered to. The analysis was conducted in accordance with the author’s specifications, following a statistical analysis plan.

## Results

### Maternal characteristics

Comparison between VCB and VBB cohorts revealed significant differences in maternal age, prevalence of high-risk pregnancies, gestational age at birth, and birth weight, as shown in Table 1.

### Fetal and neonatal short-term outcomes

Table 2 shows the results of comparison of fetal and neonatal short-term outcomes in VBB and VCB.

**Table 2:** Comparison of fetal und neonatal short-term outcomes

Comparison of fetal und neonatal short-term outcomes		VBB		VCB		*p* value	Effect size
		(*n*)	(%) or Mean ± SD median	(*n*)	(%) or Mean ± SD median		
Base excess (arterial)			− 6.37 ± 3.98 − 6.2		− 4.55 ± 3.38 − 4.4	< 0.0001	*r* = − 0.28
pH value (arterial)			7.21 ± 0.10 7.21		7.26 ± 0.09 7.26	< 0.0001	*r* = − 0.31
APGAR 1′			7.88 ± 1.93 9		8.89 ± 0.82 9	< 0.0001	*r* = − 0.34
APGAR 5′			9.25 ± 1.30 10		9.79 ± 0.59 10	< 0.0001	*r* = − 0.25
APGAR 10′			9.70 ± 0.90 10		9.93 ± 0.34 10	< 0.0001	r = − 0.13
Volume replacement	Yes	38	2.9%	1030	0.3%	< 0.0001	*V* = 0.03
Blood gas buffering	Yes	11	0.8%	395	0.1%	< 0.0001	*V* = 0.01
Mask ventilation	Yes	135	10.3%	5305	1.5%	< 0.0001	*V* = 0.04
Transfer to children’s clinic	Yes	147	10.2%	22,837	5.4%	< 0.0001	*V* = 0.01
Stillbirth (deceased subpartal)	Yes	0	0.0%	15	< 0.01%	> 0.9999	
Death 7d p.p	Yes	0	0.0%	56	< 0.01%	> 0.9999	
Duration of birth (in hours)			6.49 ± 5.48 5		5.71 ± 5.23 5	< 0.0001	*r* = 0.1

*SD* standard deviation, *VBB* vaginal breech birth, *VCB* vaginal cephalic birth

#### Mortality

There were no significant differences in mortality rates between the cohorts, both during the perinatal period (*p* = 1.0) and within the first seven days postpartum (*p* =  > 0.9999).

#### Arterial base excess and pH

Significant differences with moderate-effect sizes were observed between the breech and vertex delivery cohorts in arterial cord blood gas analysis. Arterial pH values were significantly lower in the VBB group compared to the VCB group (*p* =  < 0.0001). Similarly, arterial base excess values were significantly lower in the VBB group compared to the VCB group (*p* =  < 0.0001).

#### APGAR scores

Mean APGAR scores were significantly lower in the breech cohort compared to the vertex cohort. This was observed at 1 min (*p* =  < 0.0001), 5 min (*p* =  < 0.0001), and 10 min (*p* =  < 0.0001). Effect sizes were moderate to low.

#### Neonatal resuscitation

All measures for neonatal resuscitation and stabilization were significantly more frequently required in the breech cohort compared to the vertex cohort, albeit with low effect sizes. These included volume substitution (*p* =  < 0.0001), buffering (*p* =  < 0.0001), and mask ventilation (*p* =  < 0.0001).

#### Transfer to pediatric clinic

Significant differences were observed in the rate of transfer to pediatric clinics, with the breech cohort being significantly more frequently transferred compared to the vertex cohort (*p* =  < 0.0001). However, the effect size was extremely weak.

#### Duration of labor

The mean duration of labor (in hours) significantly differed between the groups, albeit with low effect size (*p* =  < 0.0001). It was significantly longer in the breech cohort compared to the vertex cohort.

### Maternal short-term outcomes

Table 3 shows the results of comparison of maternal short-term outcomes in VBB and VCB.

**Table 3:** Comparison of maternal short-term outcomes

Comparison of maternal short-term outcomes		VBB		VCB		*p* value	Effect size
		(*n*)	(%)	(*n*)	(%)		
Labor augmentation	Yes	923	64.3	113,213	26.8	< 0.0001	*V* = 0.05
Episiotomy	Yes	400	27.9	48,742	11.5	< 0.0001	*V* = 0.03
Perineal Tear	Yes	453	31.6	184,687	43.8	< 0.0001	*V* = 0.01
Obstetric anal sphincter injury	Yes	26	2.6	237	2.4	0.8227	
Postpartum hemorrhage > 1000 ml	Yes	33	2.3	8,193	1.9	0.3757	
Hysterectomy/laparotomy	Yes	≤ 4	/	84	0.0	0.6924	
Postpartum complications in need of treatment	Yes	9	0.6	5,401	1.3	0.0375	*V* < 0.01

*VBB* vaginal breech birth, *VCB* vaginal cephalic birth

#### Labor augmentation

A significant difference was observed between the VCB and VBB cohorts in the administration of labor-inducing agents, albeit with low effect size (*p* =  < 0.0001).

#### Episiotomy

The episiotomy rate was significantly higher in the VBB cohort compared to the VCB cohort, albeit with low effect size (*p* =  < 0.0001).

#### Perineal tears

The VBB cohort exhibited a significantly lower rate of overall perineal tears compared to the VCB cohort (*p* =  < 0.0001). However, the effect size was extremely weak. There was no significant difference in the occurrence of obstetric anal sphincter injury (third- and fourth-degree tears) (*p* = 0.8227).

#### Postpartum hemorrhage

No significant difference was observed between the VBB and VCB cohorts in the occurrence of PPH (*p* = 0.3757).

#### Hysterectomy

The rate of hysterectomy did not differ significantly between the cohorts (*p* = 0.6924).

#### Postpartum complications

The incidence of postpartum complications requiring treatment was significantly higher in the VCB cohort compared to the VBB cohort (*p* = 0.0375). However, the effect size was extremely weak.

## Discussion

### Confounding

Although significant differences were identified, which should be considered when interpreting the variables and the influence of confounders on them, mean maternal age, mean gestational age, and mean birth weight in both cohorts are within the physiological and low-risk range (see Table 1). When considering high-risk pregnancies or the presence of findings in the maternal health record, a significantly higher proportion is observed in the cephalic presentation group.

### Neonatal outcomes

#### Mortality (stillbirth and until 7 days p.p.)

The stillbirth rate showed no significant differences between the VCB and VBB groups in this study. It is important to note that the low case numbers could affect this comparison’s reliability. Dohbit et al. found no significant differences in stillbirth rates between the two groups (*p* = 0.128) [12], and Ekeus et al. observed a rate of 1.1/1000 in the vaginal breech group versus 0.1/1000 in the vaginal cephalic group, though no statistical tests were conducted to confirm the significance of these findings [13].

The mortality rate in the first seven days postpartum also showed no significant difference in this cohort. Bjellmo et al. revealed higher neonatal mortality following VBB (OR 3.0) [14]. Ekeus et al. reported neonatal mortality rates of 2.8/1000 up to 6 days postpartum and 0.6/1000 up to 28 days postpartum for the breech group, compared to 0.1/1000 for the cephalic group [13]. In a follow-up case–control study, Bjellmo et al. identified potentially preventable causes in twelve out of 31 neonatal deaths, with seven linked to complications from breech presentation [15].

#### Neonatal blood gas values and APGAR scores

In this study, arterial pH and base excess values were significantly lower in the breech cohort compared to the cephalic group, with moderate-effect sizes. While these values are objective markers of fetal oxygenation, they offer limited predictive value for short- or long-term outcomes and are not routinely used internationally [16–18]. In Germany, however, they are standard practice for initial neonatal assessment [19]. Further studies are warranted to determine the rate of neonates with pH values < 7.0 [20]. Given the absence of comparative literature and the small clinical relevance of the observed differences, these findings should be interpreted with caution.

Similarly, mean APGAR scores were lower in the breech group, particularly at 1 min, with the difference decreasing over time. The effect sizes were small to moderate. Although other studies report similar trends [12, 13, 21], this study does not allow for detailed comparison of management-related influences on these outcomes. Importantly, the predictive value of low 1-min APGAR scores is limited, while lower scores at 5 and 10 min may be more indicative of neonatal compromise [22, 23].

#### Resuscitation

In this study, measures for resuscitation and stabilization of newborns were required significantly more often in the VBB cohort compared to the VCB cohort, although the effect size of these results is extremely low. Unfortunately, no comparable results could be identified in the current literature.

#### Transfer to the pediatric clinic

Neonates from the VBB group were transferred to children’s hospitals more frequently than those from the VCB group. This also has a low effect size. No comparable outcomes were found in current research.

#### Duration of labor

The average duration of labor significantly differs between groups, with the VBB group experiencing longer durations compared to the VCB group. Dohbit et al. observed a significantly higher rate of prolonged labor (defined as > 12 h from 4 cm cervical dilation) in the VBB group compared to cephalic births (OR 8.05; *p* < 0.001) [12]. However, Nesheim et al. found that the fetal position does not affect labor duration [24]. Perl et al. found that the risk of neonatal trauma and lower Apgar scores in VBB is significantly linked to the duration of the first stage of labor, not the second. They noted that a short first stage up to 6 h or a cervical dilation rate of at least 1 cm/h is associated with a very low risk of fetal trauma. No upper limit for the second stage duration correlated with lower trauma risk, suggesting a need to reevaluate assumptions about breech birth processes and management [25].

### Maternal outcomes

#### Labor augmentation

The study identified a significantly higher rate of labor augmentation medication administered during birth in the breech presentation group compared to the cephalic presentation group, though the effect size of this finding is minimal. The increased rate of labor augmentation medication administration may be related to the practice of shortening the second phase of labor to minimize the risks for the fetus during this stage. In contrast, Dohbit et al. found no significant difference in the use of labor augmentation medications between cephalic and breech presentation births (*p* = 0.3882) [12]. Considering the risks associated with labor augmentation medication, clear medical indications should be established, especially given the lack of evidence-based recommendations for breech presentation births [26].

#### Episiotomy and perineal tears

This cohort study identified a significantly higher episiotomy rate in breech presentations compared to cephalic presentations, though the effect size is minimal. Results partially align with the existing literature; Leborne et al. observed a similar trend (*p* = 0.0012) [27], while Dohbit et al. found no significant difference (*p* = 0.301) [12].

Lower rates of perineal tears were noted in the breech group despite their higher maternal age. Studies by Fischbein et al. also reported lower risk of perineal tears but higher risk of third- and fourth-degree tears in breech births [21]. Dohbit et al. and Leborne et al. found no significant difference in the rate of perineal tears [12, 27].

Bogner et al. found that perineal injuries in supine births were mostly linked to routine episiotomies. This likely due to interventions, not just birth position. Avoiding episiotomies did not worsen neonatal outcomes [28]. This study’s high episiotomy rate, despite low overall perineal injuries, would suggest many births in the supine position, though this could not be confirmed with the data.

#### Postpartum hemorrhage and hysterectomy

No significant difference was observed in the incidence of PPH and the rate of hysterectomies between the groups. These findings are consistent with the existing literature; Dohbit et al. also found no significant differences in postpartum bleeding (> 500ml) (*p* = 0.0305), and the rate of uterine atony between VCB and VBB groups (*p* = 0.8368) [12]. Fischbein et al.’s study on out-of-hospital breech births also found no significant difference in average blood loss between VBB (314 ml) and VCB (386 ml) births (*p* = 0.15) [21].

#### Postpartum complications requiring treatment

The rate of postpartum complications requiring treatment was significantly higher in the VCB group compared to the VBB group, though this finding has a minimal effect size. No comparable results were found in the available literature.

### Influencing factors on the safety of breech birth

Factors influencing the safety of breech delivery include birth positioning, which impacts the risk of perineal trauma, with upright positions being advantageous for reducing risks and improving outcomes for both mother and child [28, 29, 30]. National guidelines from various countries emphasize the safety of vaginal breech birth depends primarily on proper risk selection and the birth setting. The birth should occur in a specialized clinic with appropriate infrastructure, including anesthesiology and pediatric emergency services. It is crucial to have specially trained and experienced midwives and obstetricians to ensure a low-risk birth with positive perinatal outcomes [6, 7, 15, 20, 31–34]. Experience depends on their self-assessed ability rather than the views of others or actual experience [31]. Furthermore, informed and participative decision-making improves maternal satisfaction and safety [35]. Finally, fetal dispositions, such as neurodevelopmental delays, could influence outcomes independent of birth circumstances and warrant further research [14, 36, 37]. Thus, fostering a comprehensive and interdisciplinary approach could enhance the safety and efficacy of vaginal breech births. Furthermore participatory decision-making during pregnancy helps reduce conflicts and anxiety, improves satisfaction with the birth experience, and ensures safety when choosing the mode of delivery [16]. Although these factors could not be directly assessed in our study, we include them here to provide a broader context for interpreting our findings and to clarify the limitations of our dataset.

### Limitations

Limitations of this study include the heterogeneity in parameters examined concerning breech delivery outcomes, lack of standardized core outcome measures, and varying definitions, leading to incomparable cases within and across studies. Retrospective data analysis risks data quality due to potentially incomplete records, unaccounted confounders like staff training, and limited insights into causalities, as databases primarily capture basic events without contextual factors [38]. The disconnection between the perinatal and neonatal datasets further complicates tracking cases over time, making it impossible to determine long-term outcomes comprehensively. As the dataset does not indicate the intended mode of delivery, outcomes of vaginal breech births that were planned but resulted in c-sections (about 30% unplanned or emergency c-sections) could not be accounted for. This limits the ability to fully assess the effectiveness of prenatal risk stratification and planned care pathways. Vaginal breech extractions—such as manual assistance—could not be separated from spontaneous vaginal breech births due to limitations of the dataset and were therefore included in the analysis. In contrast, vacuum or forceps deliveries in cephalic presentations were excluded. This and potential selection biases, along with low effect sizes, underscore the need for cautious interpretation [39, 40]. The scarcity of recent literature and the limited quality of certain studies further impact contextualization and interpretation. Another limitation is the lack of evaluation of clinically significant thresholds and proportions, which would have added further clinical relevance to the interpretation of group differences.

## Conclusion

In conclusion, this evaluation supports the effectiveness of the current system in aligning the mortality outcomes of successful vaginal breech birth (VBB) with those of successful vaginal cephalic birth (VCB). Rigorous risk stratification, education, participatory decision-making, and expert management by gynecologists and midwives have proven to be key factors in achieving these results. While mortality rates are comparable, the findings highlight an increased need for immediate neonatal care following VBB, underscoring the importance of ensuring adequate resources and preparedness for postnatal support. Similar maternal outcomes reinforce the current approach. Additionally, the mode of delivery is highly significant—not only for the birthing experience and the autonomous decision-making of the woman in labor but also for its impact on future pregnancies. Since the initial mode of delivery affects future pregnancy risks, vaginal birth should not be rejected for a c-section without well-founded reasons. Continued research on outcomes under existing clinical protocols can inform future refinements in breech birth care, with the aim of promoting safe, evidence-based practices and optimal results for mothers and newborns. These findings can also be integrated into consultations about birth options to support informed and autonomous decision-making for pregnant individuals with breech presentation.

## Data Availability

The raw data can be provided upon request to the corresponding author.
